# Tool Embodiment: The Tool’s Output Must Match the User’s Input

**DOI:** 10.3389/fnhum.2018.00537

**Published:** 2019-01-11

**Authors:** Veronica Weser, Dennis R. Proffitt

**Affiliations:** Department of Psychology, University of Virginia, Charlottesville, VA, United States

**Keywords:** tools, rubber hand illusion, embodiment, body representation, expertise

## Abstract

The embodiment of tools and rubber hands is believed to involve the modification of two separate body representations: the body schema and the body image, respectively. It is thought that tools extend the capabilities of the body’s action schema, whereas prosthetics like rubber hands are incorporated into the body image itself. Contrary to this dichotomy, recent research demonstrated that chopsticks can be embodied perceptually during a modified version of the rubber hand illusion (RHI) in which tools are held by the rubber hand and by the participant. In the present research, two experiments examined tool morpho-functional (tool output affordance, e.g., precision grasping) and sensorimotor (tool input, e.g., precision grip) match as a mechanism for this tool-use dependent change to the body image. Proprioceptive drift in the RHI occurred when the tool’s output and the user’s input matched, but not when this match was absent. This suggests that this factor may be necessary for tools to interact with the body image in the RHI.

## Introduction

Two bodies of literature have run in parallel for nearly two decades: tool use and body representation. Both fields employ overlapping terminology and examine the ways in which non-corporeal tools or prosthetics are incorporated into and extend bodily representations. Though there has been some effort to compare the two areas of research from a speculative standpoint, only minimal headway has been made to bridge the literatures experimentally. On one hand, the investigation of human tool use demonstrates that tools are incorporated into at least some form of representation of the user’s body. However, researchers who use multisensory bodily illusions like the rubber hand illusion (RHI) (Botvinick and Cohen, 1998) to examine bodily representations have repeatedly shown that the feeling of body-ownership can be extended only to objects that resemble human body parts. Thus research on tools and research using rubber hands stand in direct opposition, with many arguing that rubber hands are incorporated into the body, while tools merely extend the body. The research presented herein investigates whether consistency between the affordance of a tool and the grip used to wield it might facilitate the incorporation of a tool into the body representation in a manner akin to the rubber hand in the RHI.

In an effort to experimentally reconcile the division in the tool and body-ownership illusion literatures, Weser et al. (2017) used a novel RHI paradigm in which both the participant and the rubber hand were equipped with tools. In the classic RHI, simultaneous visuo-tactile stimulation of a rubber hand and the participant’s hidden hand induces feelings of ownership of the rubber hand (Botvinick and Cohen, 1998; Tsakiris and Haggard, 2005; Tsakiris, 2016). Importantly, the illusion also results in a change in the felt position of the hand undergoing stimulation known as proprioceptive drift: stronger subjective ownership of the rubber hand coincides with the feeling that the participant’s real hand is located closer to the rubber hand. In Weser et al. (2017), it was not the rubber hand and the participant’s hand that received simultaneous tactile stimulation, but rather the tools held by both. The unseen stimulation of the held tool is readily detected by the participant, and indeed research has shown that tactile stimulation of a tool is subjectively felt at the tip of the tool (Yamamoto and Kitazawa, 2001; Yamamoto et al., 2005), even though the mechanoreceptors that process the tactile information are located in the hand. Moreover, Miller et al. provided strong evidence that tools function as sensory extensions of the body in a manner akin to animal whiskers by showing that humans can accurately identify the location where a held rod contacts external object (2018). Of particular relevance to the present work, participants were able to identify the location of the tactile contact with the held object even when the stimulation was delivered by the experimenter (Miller et al., 2018). This indicates that the visuo-tactile stimulation delivered to the tip of the tool in the tool-version of the RHI was perceived accurately in Weser et al. (2017) and in the present work. Previously, the illusion was successfully induced when the tool in question was a pair of chopsticks, but not when it was a teacup. Moreover, the proprioceptive drift was greater for participants who practiced using chopsticks immediately prior to experiencing the illusion than for those who did not. Remarkably, the proprioceptive drift also increased as a function of chopstick skill, such that those who were highly skilled with chopsticks tended to perceive their hand as even closer to the location of the rubber hand than those who were less skilled.

The facilitatory effect of tool use prior to the illusion induction is in keeping with the literature that examines action-specific body representations. However, this finding is also at odds with the majority of the literature demonstrating a strict separation between body image and schema, representations for perception and action.

The success of the traditional RHI is contingent on the visual similarity, postural congruency, body part identity and laterality of the seen object and the body part receiving tactile stimulation (e.g., Costantini and Haggard, 2007; Haans et al., 2008). Perceived ownership of the rubber hand arises due to the modification of the body image, an abstract body representation that persists through time and contains a reference description of the visual, anatomical, and postural properties of the body (De Preester and Tsakiris, 2009). This body representation would appear to be the polar opposite of the ever-changing representation of the body’s position in space that is easily modified to include a handheld tool—the body schema. To reiterate, the body schema is what allows a tool wielder to account for the changes in his or her capacity for action during tool use, such as the longer reach afforded by a mechanical grabber. In contrast, the body image is what allows a person to recognize and identify with his or her own hand when for example, it is entwined with the hand of another. These two body representations are at the heart of each of the literatures on tool use and multisensory illusions of body ownership (e.g., RHI), respectively.

Comparing and contrasting RHI illusion and tool use work provides further support for a division between body representations for action and perception. Even though participants report feeling as if the rubber hand has become a part of their body, the reaching actions of participants who experience proprioceptive drift following the RHI remain accurate (Kammers et al., 2009). In other words, even though they report feeling as though their hand is located closer to the rubber hand, they can still accurately reach and grasp an object with the hand that was supposedly replaced by the rubber hand during the illusion. This suggests that the RHI is only modifying the perceptual representation of the body, as movements executed by the replaced hand are still accurate. This finding can be directly contrasted with work on tool use paradigms that demonstrate using tools will alter the kinematics of reach to grasp movements (Cardinali et al., 2011, 2012; Baccarini et al., 2014).

Moreover, tools have a similar null effect on the perceptual body image representation. Cardinali et al. (2011) demonstrated that the use of a reach-extending tool increases participants’ indirect length estimates of their forearms, but only when the body schema was accessed to provide the estimates. In this study, participants used a 40 cm mechanical grabbing device to reach for, grasp, lift up, and replace an object. Participants then localized one of three positions on their arm (the tip of the index finger, the wrist or the elbow) by naming the position on a scale that represented the length of the arm in response to a cue from an experimenter that was either delivered verbally (by naming either finger, wrist, or elbow) or through direct tactile stimulation of the body part. The tool-using arm was kept out of the participant’s sight behind a barrier throughout the experiment. Cardinali found that after tool use, participants overestimated the distance between their wrist and elbow if the body part was *touched* but not *named*. In contrast, localizing named body parts was not affected by tool use, suggesting that using a tool may change the body representation for action; the body schema, but not necessarily a more abstract understanding of the relative location of body parts contained in the body image (Cardinali et al., 2011). Since the work by Weser et al. (2017) represents a first attempt at using the RHI paradigm to examine whether or not the multisensory stimulation of held tools is sufficient to alter the body image as assessed by measuring proprioceptive drift, it is imperative to discover the necessary conditions and constraining factors of the tool version of the RHI. One possible mechanism is that presence or absence of morpho-functional and sensorimotor match for each tool.

In Weser et al. (2017) chopsticks and teacup were selected as comparison tools because they have a different form of morpho-functional tool output and an identical sensorimotor grip input (Cardinali et al., 2016). Chopsticks, but not teacups, have a morpho-functional match: they afford precision grasping and they are wielded with a precision grip. Teacups are typically held with a precision grip, but their morphology and function are not related to precision actions. Morpho-functional refers to the output of the tool: its shape and the action it affords. Sensorimotor refers to the input provided to the tool: the motor actions of the wielder. The match (or lack thereof) between tool morphology (output) and arm movement/grasp mechanics (input) is thought to play a deterministic role in how and whether or not the use of a tool will cause a modulation of the wielder’s body representation (Miller et al., 2014; Cardinali et al., 2016).

Broadly speaking, tools that extend one’s reach (such as mechanical grabbers) influence the wielder’s representation of the length of his or her arm, but not the size of his or her hand (Cardinali et al., 2009a,b; Miller et al., 2014). In contrast, tools that expand the grasp of the hand but not the length of the wielder’s reach specifically alter the implicit representation of the size of the hand, but not the length of the arm (Miller et al., 2014). In addition to tool type, tool dimensions and the particular movements used by a wielder also determine how the representation of the body is affected: Sposito et al. (2012) found a change when a 60 cm grabber was used, but not when a 20 cm tool was used instead. In addition, Romano et al. (2018) demonstrated that using the same tool with either predominantly shoulder movements or predominantly wrist movements determined whether participants estimated the midpoint of their forearm to be closer to their shoulder or their wrist, respectively. This finding demonstrates that tools specifically alter the representation of the body part that they are functionally augmenting, that there are particular tool properties that determine whether or not this alteration takes place, and that the way a given tool is used in a task directly affects how the body representation is updated. These studies examine long reach extending tools, or gross whole-hand grasping tools; they do not speak to whether or not small precision tools also alter the representation of the hand in a manner specific to the grip used to wield the tool (i.e., precision vs. power grips). While the difference between reach-lengthening and grip-widening tools and how they affect body representations may seem obvious, additional finer-grained comparisons as in Romano et al. (2018) are still needed to assess whether it is the shape of the tool or the grip and movements used to wield the tool that has the greater impact on the body representation.

In other words, the morpho-functional output of a tool has a clear impact on the effect that tool has on one’s body representation, but the sensorimotor input of the grip used to wield the tool and whether or not that input matches the type of action or output the tool affords must be investigated by comparing small handheld tools wielded with varying grips. Cardinali et al. (2016) found that sticks attached to the thumb and index finger and pliers both cause an increase in the represented length of the wielder’s fingers. However, the pliers caused a global increase in finger length while the two sticks specifically lengthened the representation of the wielder’s thumb and index finger. Even though both tools offered a precision output, the sensorimotor input to the tools differed. The power grip input for pliers caused the representation of the hand to shift to one where only the fingers as a unit moving in opposition to the thumb (similar to the two prongs of the pliers) were relevant. However, the precision grip input for using the sticks kept the middle, ring and pinkie finger separable from the index finger.

Cardinali et al. (2016) controlled for the morpho-functional characteristics of the tools (both had a precision output) and determined that the difference in sensorimotor input for wielding the tool affected how the hand came to be represented following use. Weser et al. (2017) controlled for the sensorimotor aspect of the tools (both had a precision grip input) and revealed that the tool without a morpho-functional and sensorimotor match (precision grip input and precision output) did not affect the body representation of the wielder. Thus, experiments that compare tools by controlling for either morpho-functional tool output or sensorimotor tool input provide a promising avenue for investigating the conditions necessary for the extension or incorporation of tools into body representations.

Experiments 1 and 2 presented herein expand on this premise by using the tool-version of the RHI to compare two tools that differ in their morpho-functional characteristics and are identical in their sensorimotor traits, as in Cardinali et al. (2016). Needle-nose pliers and tweezers both have a precision output, but the inputs differ. Needle-nose pliers are used with a whole-hand power grip while tweezers are wielded with only the thumb and index finger in a precision grip. If tool morpho-functional and sensorimotor match is a constraining factor on whether or not the tool-version of the RHI succeeds, then there should be a difference between these two tools. The tweezers (Experiment 2) should result in high proprioceptive drift following the illusion while the proprioceptive drift in the case of the pliers (Experiment 1) will not significantly differ from control conditions. This finding would bring a new level of nuance to the literature on the effects of tools on body representation, as it would indicate an advantage for morpho-functional and sensorimotor match when it comes to altering the proprioceptive information about the location of a tool and the hand wielding it. It would suggest that though a match is not necessary for a modification of the hand representation to occur (see Cardinali et al., 2016), it is required for an update to be made to the model of the body’s location in space following simultaneous multisensory stimulation. This would indicate that, as in the classic RHI, match allows for more than just the extension of the body to include the tool, but also the incorporation of the tool into the body.

## Experiment 1 Pliers: Morpho-Functional and Sensorimotor Mismatch

Chopsticks and teacups were used as comparison tools in Weser et al. (2017) because the two tools have different morpho-functional outputs and identical and sensorimotor inputs. Chopsticks have a morpho-functional/sensorimotor match and teacups do not. In Experiment 1, needle-nose pliers lack this match, as they are wielded with a full-hand power grip and act on the environment in a precision-grip manner. Therefore, it follows that a pliers-version of a RHI should not be as successful as a tool-version where there is both a morpho-functional match and a sensorimotor match, such as chopsticks (Weser et al., 2017) or tweezers (Experiment 2).

### Methods

#### Participants

A total of 71 right-handed individuals (18 males; mean age: 19.0; *SD* = 1.0) participated in exchange for credit in an introductory psychology course at the University of Virginia. The data from 5 participants was lost due to experimenter error (2) and participants’ failure to follow instruction (3), leaving 66 participants. Thirty-one participants completed the tool-skill task prior to experiencing the illusion, while the remaining 35 completed the tool-skill task at the end of the study. All participants had normal or corrected to normal vision and provided written informed consent.

### Materials

#### Pliers Rubber Hand

**FIGURE 1:**
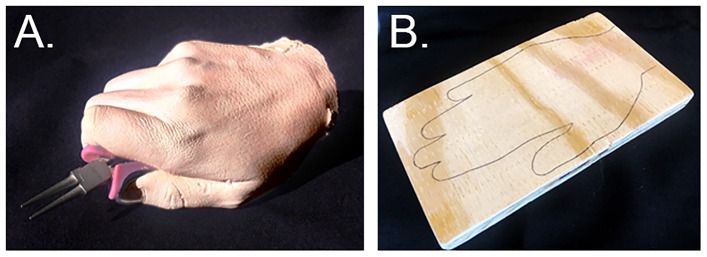
**(A)** The life cast of a hand holding needle-nose pliers. The pliers measured 13 cm in length, with a 10 cm handle and jaws 3 cm in length. **(B)** The wooden block used as the control viewed object in both experiments presented here and in Weser et al. (2017).

A life cast of author VW’s hand holding a pair of needle nose pliers was made from flesh-tinted plastic resin (see Figure 1A). An identical pair of pliers was provided for the participant to hold throughout the study and use during the tool-skill task. Together, the hand and tool measured approximately 9 cm × 20 cm × 5 cm, with the tips of the pliers resting about 2 cm above the surface of the table. The handle of the pliers contained a small spring that caused the jaws of the pliers to open whenever the user relaxed his or her grip.

#### Wooden Block

The other viewed item was the wooden block (Figure 1B) used in Weser et al. (2017). The piece of wood was a 9 cm × 23 cm × 2 cm block, pale and beige in color, with the outline of a hand drawn on the surface in black ink. This wooden stimulus was comparable in overall size to the rubber hand holding the tool, and is comparable to the control (non-corporeal) items used in classic RHI studies (i.e., Haans et al., 2008; Longo et al., 2008).

#### Tool-Skill Task

The same task used in Weser et al. (2017) was used to measure participant pliers skill. Two-hundred seventy plastic beads of various colors that measured 0.8 cm in diameter were presented to participants in a tray. Participants used their pliers to transfer each bead to a container with 6 color-labeled compartments. There were 30 beads of each color to be sorted, and 90 “distractor beads.” Participants were required to move all beads of one color to the container before starting on the next color. Participants were allotted 5 min to transfer as many beads as possible. The number of beads transferred was recorded and used as a proxy value for participant plier-skill.

#### Rubber Hand Illusion Questionnaire

Twenty-five questions from Longo et al. (2008) were adapted to measure the subjective experience of the tool-version of the RHI (see Supplementary Material for the pliers version of the questionnaire). In particular, the adapted questions referred to five different components of the experience of the illusion: embodiment of the rubber hand (10 statements), loss of the real hand (5 statements), movement of the real or rubber hand (3 statements), deafference of the real hand (3 statements), and affect (3 statements). All questions were modified to refer to the tool held by the rubber hand, rather than to the rubber hand itself.

### Experimental Design

A 2 × 2 × 2 mixed design was employed. The viewed object (pliers rubber hand vs. wooden block) and timing of visuo-tactile stimulation (synchronous vs. asynchronous) were within subjects factors, and the group (tool-skill task prior to the illusion vs. following the illusion) was a between-subjects factor. The number of beads transferred with pliers was included as a covariate, and a random effect of participant was added to account for individual differences in pliers-skill and illusion susceptibility. The 4 within-subjects conditions, completed in a random order, were: (i) pliers rubber hand synchronous (ii) pliers rubber hand asynchronous; (iii) wooden block synchronous; (iv) wooden block asynchronous. Participants completed a RHI questionnaire following the completion of each condition. Participants held pliers during all four conditions and were encouraged to stretch and rest their hand while completing the questionnaire between conditions.

In the synchronous visuo-tactile stimulation conditions, the experimenter used 2 paintbrushes to manually stroke the tip of the participant’s held pliers and the pliers held by the viewed object at the same time. In the asynchronous visuo-tactile conditions, the experimenter stroked the participant’s pliers first, while the pliers held by the viewed object was stroked with a latency of 500–1000 ms. Each stimulation period lasted 180 s and was timed using a stopwatch. Experimenters were instructed to apply enough pressure to the pliers that the contact would be felt. The paintbrush used measured 22 cm in length, with a 2 cm × 1 cm bristle.

### Procedure

**FIGURE 2:**
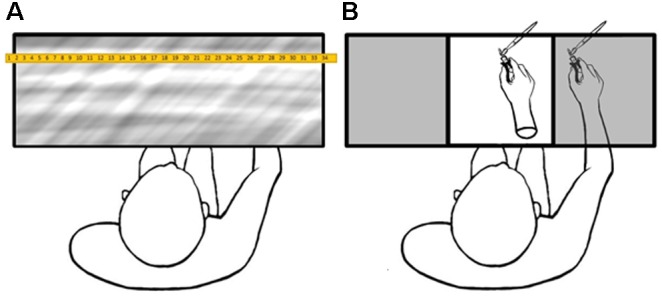
For each condition, the proprioceptive judgment phase **(A)** was conducted before and after the visuo-tactile stimulation phase **(B)**. The viewed object was visible during **(B)** and hidden during **(A)** by changing the direction of the illumination from above the surface of the mirror to below.

Participants were greeted and informed that they would be using pliers and making self-perception estimates throughout the duration of the experiment. Upon arrival, participants were randomly assigned to either first complete the tool-skill task or to undergo the RHI procedure prior to using the pliers to transfer beads. During the RHI procedure, participants were seated across from the experimenter with their right, pliers-holding hand placed inside a specially constructed box, measuring 100 cm in width, 40 cm in height, and 20 cm in depth. The box was divided into three compartments of equal size, and the viewed object rested inside the central compartment in front of the participant’s midline. The viewed object and the participant’s hand were aligned such that both rested at the same distance in front of the participant’s chest. The lateral distance between the tip of the participant’s pliers and the tip of the pliers held by the rubber hand was kept constant at 25.5 cm. The top of the box was covered by a one-way mirror. The portion of the one-way mirror above the compartment containing the participant’s hand was obstructed such that the interior of the compartment could not be seen by the participant at any time during the experiment, and the surface always appeared to be a regular, two-way mirror (Figure 2A).

The proprioceptive judgment phase was conducted before and after each visuo-tactile stimulation phase, allowing the perceived position of the participant’s held tool to be used as an implicit, quantitative proxy for measuring the strength of the illusion. A ruler with the numbers printed in reverse was supported between two poles 45 cm above the box. When illuminated from above, the mirrored surface of the box reflected the ruler numbers in their proper orientation at the same gaze depth as the floor of the box containing the rubber hand.

Before and after the visuo-tactile stimulation phase, participants verbally reported the number on the ruler that was in line with the jaws of their held pliers by projecting a parasagittal line from the tip of the tool to the ruler. During the visuo-tactile stimulation phase, the ruler was always shifted to a different random position such that the numbers the participant viewed during the judgment phases were always different. This ensured that participants did not memorize previously stated numbers and that the participant estimated the proprioceptively perceived position of their hand independently during each condition.

The central compartment of the box was illuminated from below during the visuo-tactile stimulation phase (Figure 2B), making the one-way mirror transparent such that the participant could view the stroking of the object inside the box. Throughout this procedure, participants were instructed to apply light pressure to the pliers’ handle and keep the jaws slightly closed. This allowed the experiment to stroke both jaws of the pliers simultaneously with the paint brush. During the wooden block condition, the front corner of the block (on the participant’s right) was stroked with the paint brush.

Upon completion of all four RHI conditions and the tool-skill task, participants provided a written response to a few questions about their age, sex, and a 5-point Likert question regarding their previous experience using pliers. The Likert responses ranged from: 1—I never use pliers; 2—I very rarely use pliers (e.g., I’ve used them in the last year); 3—I occasionally use pliers (e.g., I’ve used them a few times in the last 6 months); 4—I frequently use pliers (e.g., I often use them in crafts or projects); 5—I use pliers regularly (e.g., I use them once a week or more).

### Results and Discussion

#### Proprioceptive Drift

**FIGURE 3:**
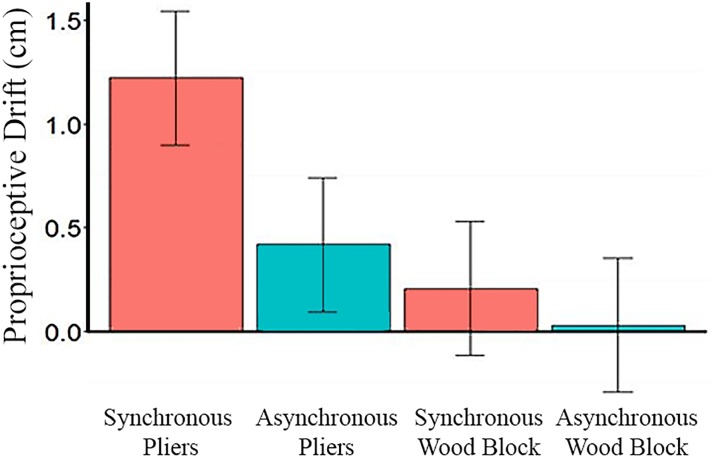
The non-significant interaction of timing of visuo-tactile stimulation and viewed object. The significant main effect of viewed object is apparent, as drift was larger when participants viewed a rubber hand holding pliers than when a wooden block was viewed. Error bars represent ±1 SEM.

As predicted, participants did not experiencea significant difference in proprioceptive drift during the synchronous stroking of their held pliers and the pliers held by the rubber hand as compared to the control conditions with asynchronous stroking and the wooden block. For this analysis, assumptions of normal distribution, independence of residuals, and sphericity were met. Using R (R Core Team, 2017) and the lmer() function in the lme4 library (Bates et al., 2014), a model was fitted to the data that predicted drift from the interaction of timing of visual-tactile stimulation (synchronous or asynchronous), viewed object (pliers rubber hand vs. wooden block), recency of tool-use (before or after the illusion phase) as between-subjects fixed effects. The amount of beads transferred during the tool skill task was included as a covariate and a random effect of participant was used to account for the repeated measures nature of the design. The main effect of viewed object was significant: Wald Chi-Square (1) = 5.46, *p* = 0.019, with the pliers rubber hand (*M* = 0.87, *SE* = 0.24) yielding significantly higher proprioceptive drift than the wooden block (*M* = 0.14, *SE* = 0.23). Most RHI studies focus on the interaction between visual-tactile stimulation and the viewed object, and as Figure 3 illustrates, this interaction was not significant (*p* = 0.33).

**FIGURE 4:**
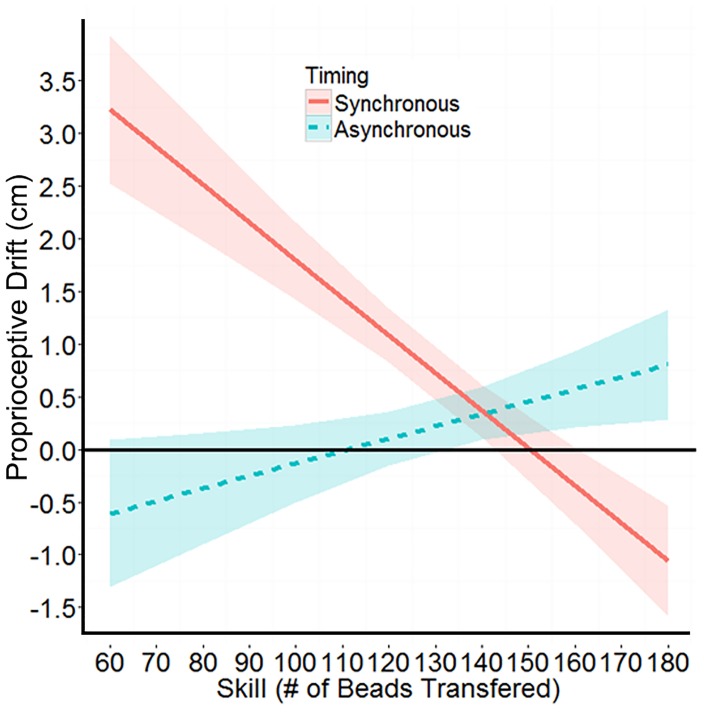
The significant interaction between the timing of visuo-tactile stimulation, and the number of beads transferred during the tool-skill task. Shaded areas indicate ±1 SEM.

There was also a significant interaction of timing of visuo-tactile stimulation (synchronous vs. asynchronous) and the number of beads transferred during the tool-skill task: Wald Chi-Square (1) = 13.92, *p* < 0.001. This interaction is plotted in Figure 4. There were no other main effects or interactions that reached significance. Clearly, this interaction was not predicted given the opposite findings with chopsticks in Weser et al. (2017); however, the pliers and chopsticks conditions differed in a number of respects. Unlike previous studies in which many participants reported that they used chopsticks daily, no participants in Experiment 1 reported frequently using pliers. Indeed, the majority of participants (*n* = 43) said they “very rarely” used pliers. Moreover, when grouping participants by their response to the pliers-use question, the 4 participants who said they “frequently use pliers” transferred the fewest beads during the took-skill task of any group (*M* = 103, *SD* = 9.2; Occasionally: *n* = 4, *M* = 161, *SD* = 11.7; Very Rarely: *n* = 43, *M* = 155, *SD* = 20.9; Never: *n* = 15, *M* = 115, *SD* = 26.5).

This suggests that the bead transfer task may not have been an ecologically valid assessment of tool skill, as it was for chopsticks. It also indicates that those performing better (e.g., transferring more beads) were not necessarily those participants with more skill and experience at using pliers. This may offer an explanation both as to why there was no effect of group (tool-skill task prior to the illusion vs. after the illusion) on the illusion, and importantly why the interaction of number of beads transferred and timing of visuo-tactile stimulation was the opposite direction as previously seen in the chopsticks study in Weser et al. (2017). In that experiment, the tool-skill task successfully quantified the tool-users’ skill with chopsticks, as participants who reported more frequent chopstick use far outperformed those who reported never or infrequently using the tool. It is therefore a possibility that transferring beads with pliers was not so much a measure of skill with pliers, but rather of overall hand dexterity.

Though as of yet there is no definitive experiment that demonstrates a decrease in RHI strength for those with greater hand dexterity or awareness (dancers or pianists, for example), it has long been speculated that such individuals would have reduced susceptibility to the illusion (Botvinick and Cohen, 1998; Tsakiris and Haggard, 2005; Tsakiris, 2010). Indeed, those with lower interoceptive awareness (as measured with an established heart-rate monitoring task) were far more susceptible to the RHI than were those with high interoceptive abilities (Tsakiris et al., 2011; but see David et al., 2014). Therefore, the strong negative relationship seen in this study between the number of beads transferred with pliers and the amount of proprioceptive drift experienced during synchronous illusion conditions may actually index the decreased illusion susceptibility of more dexterous, bodily aware participants who are able to use an unfamiliar tool more easily than participants with less bodily awareness.

#### Rubber Hand Illusion Questionnaire

**FIGURE 5:**
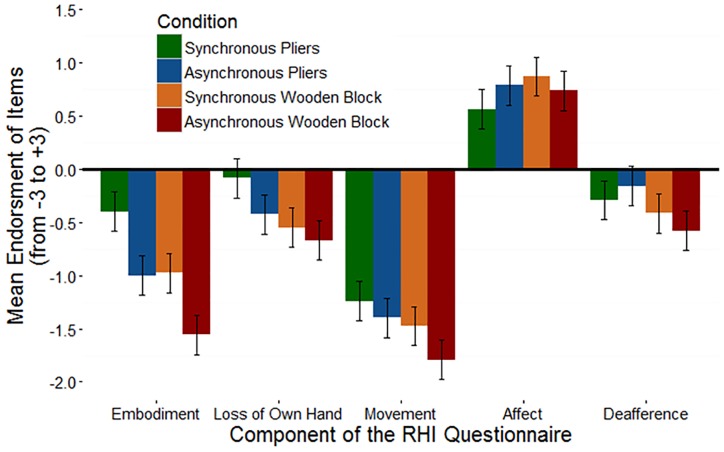
The non-significant interaction between RHI condition and questionnaire component for the pliers-version of the RHI. Error bars represent ±1 SEM.

Following Longo et al. (2008), the mean ratings for the five components of the rubber hand illusion questionnaire (Embodiment, Loss of one’s hand, Movement, Affect, and Deafference) were submitted to a mixed ANOVA with the 4 illusion conditions as within-subjects factors and a between subject factor of group (tool-skill task prior to the illusion vs. after). The analysis revealed a significant main effect of questionnaire component [*F*(4,62) = 80.47, *p* < 0.001] and of condition [*F*(4,88) = 5.92, *p* < 0.001]. No other main effects of interactions reached significance (all *F*’s > 1.0). Follow-up analyses examining each questionnaire component individually revealed no significant differences between illusion conditions, suggesting that the subjective experience of the pliers-version of the rubber hand illusion was not greatly affected by the appearance of the viewed object or by the timing of the visuo-tactile stimulation. It seems likely that participants’ lack of familiarity with pliers made it just as difficult for them to embody a rubber hand holding pliers as it would be to embody a wooden block. As a result, they failed to endorse questions about the rubber hand and the wooden block at equal rates. The non-significant interaction between illusion condition and component of the RHI Questionnaire is plotted in Figure 5.

## Experiment 2 Tweezers: Morpho-Functional and Sensorimotor Match

Like the chopsticks and teacups used in Weser et al. (2017), tweezers and pliers similarly differ on their level of morpho-functional match and sensorimotor match: Chopsticks and tweezers match, while teacups and pliers do not. Weser et al. (2017) examined tools with an identical sensorimotor match (both tools were held with a precision grip) while the studies presented here examine tools with an identical morpho-functional match (both tools act on the environment in a precision fashion). If the match between tool morphology and grasp mechanics determines whether or not the use of a tool will cause a modulation of the wielder’s body representation (e.g., Miller et al., 2014; Cardinali et al., 2016; Weser et al., 2017), then participants in Experiment 2 should experience a RHI when viewing a rubber hand holding tweezers.

### Methods

#### Participants

Data was collected from 76 right-handed participants (24 males; mean age: 18.7; *SD* = 1.0). All participants had normal or corrected to normal vision, participated in exchange for credit in an introductory psychology course at the University of Virginia, and provided written informed consent prior to commencing the study. Data from 4 female participants was lost due to experimenter error (1) and the failure of 3 participants to follow directions. This brought the total sample size down to 72, with 37 completing the tool-skill task prior to engaging in the illusion, and the final 35 completing the tool-skill task after the completion of the illusion procedure.

### Materials

#### Tweezers Rubber Hand

**FIGURE 6:**
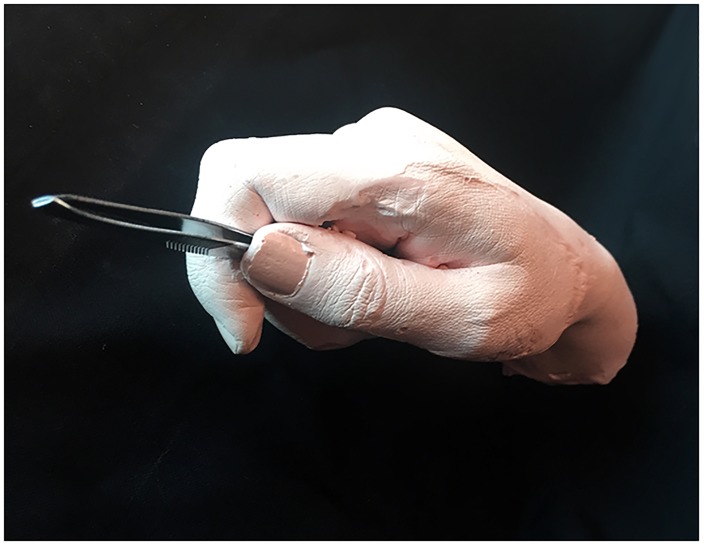
The life cast of a hand holding tweezers. The tweezers measured 9 cm in length.

A life cast of author VW’s hand holding a pair of tweezers was made from flesh-tinted plastic resin (see Figure 6). An identical pair of tweezers was provided for the participant to hold throughout the study and use during the tool-skill task. Together, the hand and tool measured approximately 9 cm × 22 cm × 6 cm, with the tips of the tweezers resting about 2 cm above the surface of the table.

#### Wooden Block

The other viewed item was the wooden block (Figure 1B) described in Experiment 1.

#### Tool-Skill Task

The bead-transfer task described previously was altered so that it would be more appropriate for tweezers. The beads were replaced with “seed beads,” tiny plastic beads that measured 1.8 mm in diameter. As before, participants were required to use tweezers to pick up 1 bead at a time and move it from 1 container to another, sorting by color. There were 40 beads of each of 8 colors (320 beads total), and participants were allotted 5 min to sort as many beads as possible.

#### Rubber Hand Illusion Questionnaire

The same 25 questions from Longo et al. (2008) were used. The questions were altered so as to reference the rubber hand holding tweezers, rather than the rubber hand alone or the rubber hand holding pliers.

#### Tweezers Use Question

Given our hypothesis that tool experience (or lack the thereof) played a large role in the findings presented in Experiment 1 in which most participants reported infrequent pliers-use, we decided to closely examine tweezers use in Experiment 2. As in Experiment 1, we used a brief post-experiment questionnaire assessing tool familiarity. The Likert responses ranged from: 1—I never use tweezers; 2—I very rarely use tweezers (e.g., I’ve used them to remove a splinter here and there); 3—I occasionally use tweezers (e.g., I used them here and there for splinters, projects, or personal grooming); 4—I frequently use tweezers (e.g., I use them monthly for splinters, projects, or personal grooming); 5—I use tweezers regularly (e.g., I use them once a week or more).

### Experimental Design

A 2 × 2 × 2 × 2 mixed design was employed. The viewed object (tweezers rubber hand vs. wooden block) and timing of visuo-tactile stimulation (synchronous vs. asynchronous) were within-subjects factors, the group (tool-skill task prior to the illusion vs. following the illusion), and frequency of tweezers use were between-subjects factors. The number of beads transferred with tweezers was included as a covariate, and a random effect of participant was added to account for individual differences in tweezers-skill and illusion susceptibility. The four within-subjects conditions, completed in a random order, were: (i) tweezers rubber hand synchronous (ii) tweezers rubber hand asynchronous; (iii) wooden block synchronous; (iv) wooden block asynchronous. The participant held tweezers during all four conditions of the illusion.

### Procedure

Upon arrival, participants were randomly assigned to either first complete the tool-skill task or to undergo the RHI procedure prior to using the tweezers to transfer seed beads. During the illusion-induction procedure, participants were instructed to apply light pressure to the tweezers handle and keep the prongs slightly closed. This allowed the experimenter to stroke both prongs of the tweezers simultaneously with a paint brush. All other aspects of the procedure were identical to Experiment 1.

### Results and Discussion

#### Proprioceptive Drift

We found that participants who reported frequent tweezers use experienced proprioceptive drift when their tweezers were stroked in synchrony with the tweezers held by the rubber hand. Participants who did not report frequent tweezers use did not experience proprioceptive drift that differed significantly between the synchronous tweezers condition of interest and the control conditions. Assumptions of normal distribution, independence of residuals, and sphericity were met. A linear mixed-effects model that included parameters for viewed object (tweezers rubber hand vs. wooden block), visuo-tactile stimulation (synchronous vs. asynchronous), tool skill (number of beads transferred), and recentness of tool use (tool-skill before vs. after the illusion), was fitted to the data. The model also included a random effect of participant and a between-subjects factor of response to the tweezers user question (never vs. rarely vs. occasionally vs. frequently vs. regularly). The main effect of timing of visuo-tactile stimulation was significant: Wald Chi-Square (1) = 4.85, *p* = 0.028, with synchronous stimulation (*M* = 0.24, *SE* = 0.24) yielding significantly higher proprioceptive drift than asynchronous stimulation (*M* = −0.39, *SE* = 0.24). In addition to the main effect, the interaction between viewed object, timing of visuo-tactile stimulation and tweezers use response was significant: Wald Chi-Square (1) = 10.22, *p* = 0.037.

**FIGURE 7:**
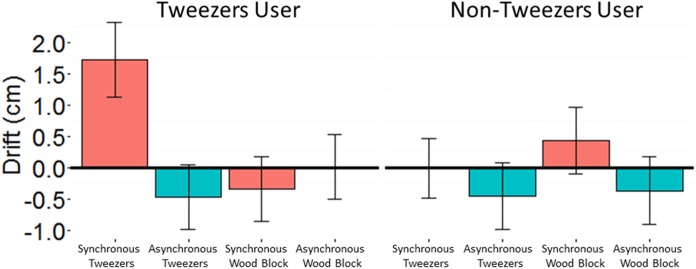
The significant interaction of viewed object, timing of visuo-tactile stimulation and the median split of self-report of tweezers-use. Error bars represent ±1 SEM.

To ease in the interpretation of this interaction, we divided participants into two categories: frequent tweezers use (*n* = 35) vs. little or no tweezers use (*n* = 37). The choice to employ a median split in our analysis was made on the basis of our desire to compare the means of two groups as a more direct method of addressing the research question: whether or not frequent use of a tool facilitates proprioceptive drift in the RHI. We fit a new model to the data identical to the model described above except that the between-subjects effect of tweezers use was dichotomous (frequent use vs. little to no use). Again the main effect of timing of visuo-tactile stimulation was significant: Wald Chi-Square (1) = 4.65, *p* = 0.031. As before, the interaction between viewed object, timing of visuo-tactile stimulation and tweezers use/little or no use was significant: Wald Chi-Square (1) = 5.57, *p* = 0.018. The interaction is plotted in Figure 7.

Although not significant, the interaction between self-reported frequent use of tweezers and tweezers skill trended in the predicted direction: Wald Chi-Square (1) = 3.19, *p* = 0.074, with frequent tweezers users transferring more beads (*M* = 146, *SE* = 24) than non-frequent users (*M* = 130, *SE* = 24). Recentness of tool use was not significant and did not interact with any of the other parameters, and no other main effects or interactions were significant (all *p*’s > 0.01). A follow-up analysis that included a factor for participant sex was conducted to ensure that the tweezers use was not sex-dependent. Indeed, sex did not significantly affect proprioceptive drift (*p* > 0.3), nor did it interact with any other factor in the model (all *p*’s > 0.25).

#### Rubber Hand Illusion Questionnaire

**FIGURE 8:**
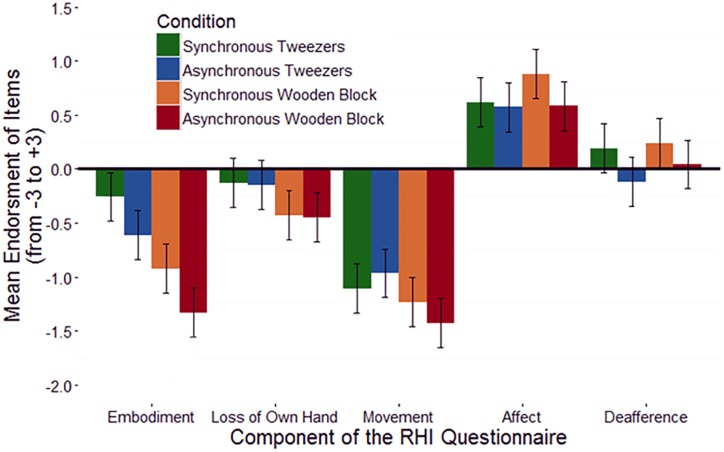
The non-significant interaction between RHI condition and questionnaire component for the tweezers-version of the RHI. Error bars represent ±1 SEM.

Following Longo et al. (2008), mean ratings for the five components of the rubber hand illusion questionnaire (Embodiment, Loss of one’s hand, Movement, Affect, and Deafference) were submitted to an ANOVA with the four illusion conditions (Tweezers Rubber Hand Synchronous, Tweezers Rubber Hand Asynchronous, Wooden Block Synchronous, and Wooden Block Asynchronous) as within-subjects factors. The ANOVA revealed a significant effect of questionnaire component [*F*(4,67) = 40.53, *p* < 0.001] and a trending effect of condition [*F*(4,68) = 2.45, *p* = 0.062]. The interaction was not significant. To follow-up this finding, an ANOVA examining differences in participants’ endorsement of Embodiment-related questions in the four conditions was conducted. This ANOVA revealed a significant effect of condition: *F*(3,68) = 3.86, *p* = 0.010, with the synchronous tweezers condition resulting in slightly more positive endorsement of embodiment items (*M* = −0.26, *SE* = 0.21) than the other conditions (asynchronous tweezers: *M* = −0.61, *SE* = 0.22; synchronous wooden block: *M* = −0.92, *SE* = 0.26; asynchronous wooden block: *M* = −1.33, *SE* = 0.24). This finding is consistent with previous studies, which similarly find a small advantage for the synchronous condition in which the viewed object matches the object held by the participant. The interaction of component of the RHI Questionnaire and the illusion condition is plotted in Figure 8.

The significant interaction between viewed object, timing of visuo-tactile stimulation and tweezers-use status adds credence to the idea that morpho-functional match and sensorimotor match is an important component for the success of the illusion, and suggests that it is only the tools that match on these dimensions (chopsticks and tweezers) that integrate sufficiently with the body representation to affect an illusion of body ownership like the RHI. Although a median split was used in the analysis, the results suggest that the illusion only succeeds for individuals who report actual experience using the tweezers on a regular basis. Chopsticks are a relatively complicated tool to use, and so only those with chopsticks experience succeed at the tool skill task. On the other hand, tweezers are very simple to use and so even participants with very little real-world tweezers experience were able to transfer many beads. Therefore, the effects of the illusion emerge when participants’ real world experience with tweezers are taken into account, rather than when examining their success at a somewhat arbitrary measure of tool-skill.

## Cross-Experiment Comparison

**FIGURE 9:**
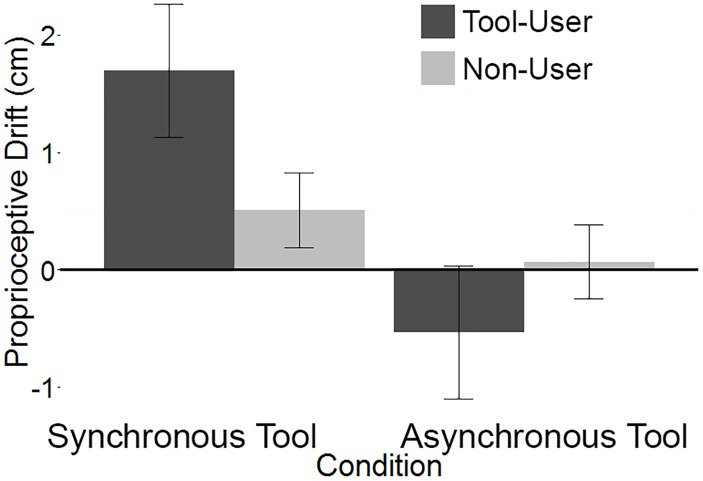
An analysis of the combined Experiments 1 and 2 data. The trending interaction suggests that across experiments, participants who self-report using the tool frequently (Pliers: *n* = 8, Tweezers *n* = 35), experience more proprioceptive drift in the synchronous visuo-tactile stimulation condition than do the participants who do not report frequent tool-use (Pliers: *n* = 58, Tweezers: *n* = 37). The effect reverses for the asynchronous condition. Error bars represent ±1 SEM.

To compare the amount of proprioceptive drift experienced by participants in the pliers and tweezers versions of the illusion, the self-reported responses to the tool-use question in Experiment 1 was similarly divided into two groups that resulted in a frequent pliers use group (*n* = 8) and a little or no pliers use group (*n* = 58). This allowed the data from the synchronous and asynchronous tool conditions from both experiments to be combined. A linear mixed-effects model that included parameters for Experiment (pliers vs. tweezers), Condition (synchronous tool vs. asynchronous tool), and self-reported tool use (tool user vs. non-user) was fitted to the data. The model also included a random effect of participant. As expected, the main effect of condition was significant: Wald Chi-Square (3) = 6.89, *p* = 0.009, with synchronous tool (*M* = 0.86, *SE* = 3.00) yielding significantly higher proprioceptive drift than asynchronous tool (*M* = −0.02, *SE* = 2.98). There was also a significant effect of Experiment, with the pliers experiment yielding significantly higher proprioceptive drift (*M* = 0.87, *SE* = 2.72) than the Tweezers experiment (*M* = 0.01, *SE* = 2.23) across both conditions and levels of self-reported tool use: Wald Chi-Square (1) = 5.83, *p* = 0.016. Finally, the interaction between condition and self-reported tool use, plotted in Figure 9, was trending toward significance: Wald Chi-Square (1) = 3.80, *p* = 0.051. No other main effects or interactions reached significance (all *p*’s > 0.3).

This finding suggests that tool experience is an important variable to consider in future investigations of the tool version of the RHI, as well as studies employing other paradigms to investigate the effects of tools on their wielders.

## Conclusion

The effects of tools and rubber hands on body representations have been reported in disparate literatures since both fields began to gain traction in the past 20 years. Similarly, for over 100 years, researchers have recognized a sharp divide between the body schema, a body representation for action, and the body image, a body representation for perception and identification (Head and Holmes, 1911; Anema et al., 2009). Many have argued that the embodiment of external limbs in the RHI is fundamentally different from the type of embodiment experienced by tool users (De Preester and Tsakiris, 2009; De Vignemont and Farné, 2010). Although both skilled tool users and individuals who experience the RHI report that the tool or rubber hand feels like it is a part of their body, the effects of tool-incorporation and rubber-hand incorporation on subsequent behavior are markedly different.

Barring brain injury or the isolated study of a particular type of embodiment through illusion or tool training studies, the body schema and the body image must work in harmony for one to experience a coherent sense of control over and identification with one’s physical form. Thus it seems likely that the two representations are not entirely separate. Weser et al. (2017) set out to examine the link between the embodiment of tools and rubber hands by adapting the classic RHI illusion to include a handheld tool. Skillfully using chopsticks prior to experiencing a RHI in which chopsticks receive the visuo-tactile stimulation increases the experience of the illusion, as measured behaviorally through proprioceptive drift (Weser et al., 2017).

Proprioceptive drift is the difference between a participant’s estimate of the position of his or her own hand before and after the visuo-tactile stimulation of the real and rubber hands. Proprioceptive drift is believed to be a behavioral measure of the RHI that indexes the effect of visuo-tactile stimulation of non-corporeal objects on the body image. Since the RHI is performed with passive tactile stimulation and measured with introspective report and visual judgments of the location of one’s hand, it is believed to be a purely perceptual (as opposed to motoric) illusion that only alters the body image, not the body schema (De Vignemont and Farné, 2010). In contrast, practice using a tool results in real time updates to one’s capacity for action that is captured by changes to the body schema, as typically measured in changes in reach-to-grasp kinematics (e.g., Cardinali et al., 2012; Baccarini et al., 2014), changes in tactile acuity suggesting a perceived lengthening of the arm or widening of the hand, depending on the type of tool used (e.g., Cardinali et al., 2011; Canzoneri et al., 2013; Miller et al., 2017), and a tendency to overestimate the length of the arm following reach-extending tool use as measured in the forearm bisection task (e.g., Sposito et al., 2012; Romano et al., 2018). The findings of Weser et al. (2017) study using chopsticks and Experiment 2 presented here were novel because the motoric changes to the body schema following chopstick use or tweezer use manifested in perceptual drift in the RHI, a purely perceptual measure of the body image. Consistent with the tool-use literature, perceptual drift was larger for participants in Weser et al. (2017) study who had a chance to use chopsticks prior to experiencing the illusion. In the present work, the finding that only frequent tweezers users experienced the illusion adds nuance to the burgeoning literature and suggests future avenues of research should solicit expert tool users as participants. Although recent or frequent use of the tool facilitated the illusion, the lack of success of Weser et al. (2017) study using a teacup as the tool held by the rubber hand indicates the mere familiarity of the tool is not enough to facilitate its embodiment in this paradigm. This suggests that even if the participants in Experiment 1 had been familiar with pliers, they likely would not have experienced significant proprioceptive drift.

Most researchers report a strong correlation between subjective reports of the experience of the illusion (e.g., “it felt like the rubber hand was part of my body”) and proprioceptive drift *toward* the rubber hand (e.g., Tsakiris and Haggard, 2005). In other words, participants estimate that their hand is closer to the rubber hand when they have a stronger feeling that the rubber hand is part of them. However, in both Weser et al. (2017) and the studies presented here, synchronous stimulation of a rubber hand holding a tool matching the participant’s own held tool did not result in high self-reported rubber hand embodiment, even when synchronous visuo-tactile stimulation did cause high proprioceptive drift. So although tool-versions of the RHI do provide evidence for cross-talk between the body models for perception and action, the introspective aspect of this perceptual illusion seems to be less susceptible to modification from tools. The studies presented here were designed to investigate the difference in the behavioral outcome of the chopsticks and teacup version of the RHI conducted in Weser et al. (2017), but together they also contribute to the mounting evidence that proprioceptive drift and the introspective questionnaires used in the RHI literature do not necessarily measure the same phenomena, as they are not always strongly or even positively correlated (e.g., Holmes et al., 2006; Makin et al., 2008; Rohde et al., 2011).

The effect of morpho-functional and sensorimotor match on the proprioceptive drift outcome of the tool-versions of the RHI was the driving force behind the studies presented here. The morpho-functional component of a tool refers to its shape and the action it affords—the tool’s output. Sensorimotor is the wielder’s actions and grip while using the tool—the wielder’s input. It has been speculated that the match or lack of match determines whether or not a modulation of the wielder’s body representation occurs (Miller et al., 2014; Cardinali et al., 2016). The experiments presented here examined the difference between tools that both act on the environment in a precision manner, and therefore have the same morpho-functional output, but the tools are operated with either a power grip (pliers) or a precision grip (tweezers), and therefore differ in their sensorimotor input. Only the tool with a morpho-functional and sensorimotor match (tweezers: precision action, precision grip) resulted in a successful tool-version of the RHI, confirming that the same match found in chopsticks may play a deciding role in the illusion’s success. That said, the tweezers version of the illusion only succeeded for participants who reported frequent tweezers use, suggesting that tool experience also effects whether or not a tool will alter one’s body representation.

When given the opportunity to use chopsticks prior to experiencing the tool-version of the RHI, the changes to the body schema manifested in increased proprioceptive drift relative to the drift experienced by individuals who used the tool following the illusion. Moreover, only individuals who frequently used tweezers experienced a tweezers version of the RHI, suggesting that long term tool use facilitated body image modification during synchronous visuo-tactile stimulation of real and rubber hands holding tools. Taken together, this indicates that the body image remains distinct from the body schema when it comes to introspective self-identification, but that taking action with tools can alter perceptual models of the body. The exploration of the mechanisms that contribute to and are responsible for tool-effects on body representations makes an important contribution to the literature: it is an investigation of the complex interplay between bottom-up effects such as simultaneous multisensory integration and tool experience with more top-down knowledge about body appearance, identity, position, tool function, appropriate grip, and tool expertise.

## Ethics Statement

This study wascarried out in accordance with the recommendations for studies involving human subjects and the protocol was approved by the Institutional Review Board for Social and Behavioral Sciences at the University of Virginia. All participants gave written informed consent in accordance with the Declaration of Helsinki.

## Data Availability Statement

The datasets generated for these experiments can be found in figshare data repository. Pliers data: 10.6084/m9.figshare.7177118 Tweezers data: 10.6084/m9.figshare.7177115.

## Author Contributions

VW conceived of the presented idea. VW and DP designed the studies. VW created the materials and collected and analyzed the data under the supervision of DP. VW and DP discussed the results and contributed to the final manuscript.

## Conflict of Interest Statement

The authors declare that the research was conducted in the absence of any commercial or financial relationships that could be construed as a potential conflict of interest.
