# Supplementary material for: Tool Embodiment: The Tool’s Output Must Match the User’s Input
Source: Front Hum Neurosci. 2019 Jan 11;12:537. doi: 10.3389/fnhum.2018.00537 (PMC6336895; doi:10.3389/fnhum.2018.00537)
Supplement: Supplementary file 1 [file Table_1.DOCX]

| **Supplementary Material “Tool Embodiment: The Tool’s Output Must Match the User’s Input”**  **Pliers Version of the Rubber Hand Illusion Questionnaire**  Ques  In the questions below, -3 corresponds to "completely disagree", while +3 corresponds to "completely agree". 0 corresponds to "neither agree nor disagree".  Please answer the following questions about your experience using the scale from -3 to +3. | | | | | | | |
| --- | --- | --- | --- | --- | --- | --- | --- |
|  | -3 | -2 | -1 | 0 | 1 | 2 | 3 |
| It seemed like I was looking directly at my own hand holding pliers, rather than at a rubber hand holding pliers. |  |  |  |  |  |  |  |
| It seemed like the pliers I was holding were in the location where the pliers were held by the rubber hand. |  |  |  |  |  |  |  |
| It seemed like the rubber hand holding pliers was moving towards my hand. |  |  |  |  |  |  |  |
| It seemed like the rubber hand holding pliers was my hand. |  |  |  |  |  |  |  |
| It seemed like I had three hands. |  |  |  |  |  |  |  |
| It seemed like the rubber hand holding pliers was part of my body. |  |  |  |  |  |  |  |
| I had the sensation of pins and needles in my hand. |  |  |  |  |  |  |  |
| It seemed like the rubber hand holding pliers was in the location where my hand was holding pliers. |  |  |  |  |  |  |  |
| It seemed like the rubber hand holding pliers belonged to me. |  |  |  |  |  |  |  |
| I found that experience interesting. |  |  |  |  |  |  |  |
| It seemed like I could have moved the pliers in the rubber hand if I had wanted. |  |  |  |  |  |  |  |
| It seemed like my own hand became rubbery. |  |  |  |  |  |  |  |
| It seemed like I was unable to move the pliers in my hand. |  |  |  |  |  |  |  |
| It seemed like my hand had disappeared. |  |  |  |  |  |  |  |
| The touch of the paintbrush on my pliers was pleasant. |  |  |  |  |  |  |  |
| It seemed like my hand was out of control. |  |  |  |  |  |  |  |
| I found that experience enjoyable. |  |  |  |  |  |  |  |
| It seemed like I could have moved the pliers I was holding if I wanted. |  |  |  |  |  |  |  |
| It seemed like my hand was moving towards the rubber hand. |  |  |  |  |  |  |  |
| It seemed like I was in control of the pliers held by the rubber hand. |  |  |  |  |  |  |  |
| It seemed like I couldn’t really tell where my hand was. |  |  |  |  |  |  |  |
| It seemed like the experience of my hands was less vivid than normal. |  |  |  |  |  |  |  |
| I had the sensation that my hand was numb. |  |  |  |  |  |  |  |
| It seemed like the touch I felt was caused by the paintbrush touching the chopsticks held by the rubber hand. |  |  |  |  |  |  |  |
| It seemed like the rubber hand began to resemble my real hand. |  |  |  |  |  |  |  |
